# Case Report: Compromised response of memory-formed bystander T cells after CD19 CAR-T cell therapy following CD20 bispecific antibody therapy

**DOI:** 10.3389/fimmu.2026.1756756

**Published:** 2026-04-22

**Authors:** Yuya Masuda, Junichi Kato, Tatsuya Konishi, Takatsugu Honda, Masaki Maruta, Natsumi Kawasaki, Meika Matsumoto, Koji Oka, Kento Mori, Shogo Nabe, Yukihiro Miyazaki, Etsuko Matsubara, Shingo Urata, Shingo Kinnami, Yasukazu Doi, Yasunori Takasuka, Jun Yamanouchi, Toshiki Ochi, Katsuto Takenaka

**Affiliations:** 1 Department of Hematology, Clinical Immunology, and Infectious Diseases, Ehime University Graduate School of Medicine, Toon, Ehime, Japan; 2 Division of Blood Transfusion and Cell Therapy, Ehime University Hospital, Toon, Ehime, Japan; 3 Department of Clinical Laboratory, Ehime University Hospital, Toon, Ehime, Japan; 4 Department of Internal Medicine, Matsuyama Red Cross Hospital, Matsuyama, Ehime, Japan; 5 Division of Immune Regulation, Proteo-Science Center, Ehime University, Toon, Ehime, Japan

**Keywords:** Adenoviral cystitis, bispecific antibody (BsAb), bystander T cells, chimeric antigen receptor (CAR)-T cells, diffuse large B-cell lymphoma (DLBCL)

## Abstract

T-cell redirection using chimeric antigen receptor (CAR)-T cells and/or bispecific antibody (BsAb) has been recognized as a new therapeutic option for relapsed/refractory large B-cell lymphoma. Bystander T cells in the body can affect immune responses after the treatment; however, their memory T-cell characteristics and antigen-specific responses still remain undefined. Here, we focused on memory-formed bystander T cells, and considered their viral-specific responses in a relapsed/refractory diffuse large B-cell lymphoma patient who developed adenoviral hemorrhagic cystitis as an uncommon complication after CD19 CAR-T cell therapy following CD20 BsAb therapy. After BsAb therapy, effector memory T cells were the predominant population in the patient’s peripheral blood, achieving a complete response without unwanted viral infection. Although memory T-cell phenotypes remained unchanged for 8 weeks after pausing of BsAb therapy, it was found that bystander central/effector memory T cells had newly and successfully developed in the peripheral blood after sequential CAR-T cell therapy. Importantly, this patient did not develop any common viral infections during the course of treatment, such as reactivation of cytomegalovirus; however, adenoviral cystitis occurred even in the presence of memory T cells in the periphery after CAR-T cell therapy but not during BsAb therapy. These findings appear to suggest a difference between memory T-cell responses during BsAb therapy and those following CAR-T cell therapy, providing an important opportunity to reconsider memory-formed but newly-developed bystander T cells and their antigen-specific T-cell responses after CAR-T cell therapy.

## Introduction

T-cell redirection such as the use of chimeric antigen receptor (CAR)-T cells and/or bispecific antibody (BsAb) has been recognized as a new therapeutic option for relapsed/refractory large B-cell lymphoma (1, 2). CD19 CAR-T cells, such as tisagenlecleucel (tisa-cel), axicabtagene ciloleucel (axi-cel), and lisocabtagene maraleucel (liso-cel) as a gene-modified T-cell product can induce overall response rates (ORRs) of around 50-80% against diffuse large B-cell lymphoma (DLBCL) with a single dose (1, 3–6). In contrast, BsAbs targeting CD20 and CD3, such as epcoritamab, mosunetuzumab, and glofitamab as off-the-shelf modalities, have shown ORRs of around 50% against DLBCL; however, repetitive administration is often required in order to maintain their therapeutic effects (2, 7, 8). As the two modalities are mutually complementary, sequential approaches using CAR-T cells and BsAbs are planned to further enhance their anti-lymphoma effects. Indeed, BsAb therapy has been reportedly effective both before and after CAR-T cell therapy (7, 9, 10). Since autologous T cells are basically employed for this type of treatment, there is no need for systemic immunosuppressant medication other than temporary administration of tocilizumab and corticosteroids to control cytokine-release syndrome (CRS) and immune effector cell-associated neurotoxicity syndrome (ICANS) (11). Based on this, viral infections that can occur as a result of normal B-cell suppression and hypogammaglobulinemia after CD19 CAR-T cell/CD20 BsAb therapy have been extensively discussed; however, the characteristics of bystander memory T cells (irrelevant T cells such as non-CAR-T cells as well as tumor-unrelated T cells) (12) and their responses after CAR-T cell/BsAb therapy remain to be clarified. Here, we focused on memory-formed T cells after CD19 CAR-T cell therapy following CD20 BsAb therapy, and considered their antigen-specific responses in a unique patient who developed adenoviral hemorrhagic cystitis as an uncommon complication after CD19 CAR-T cell therapy but not during CD20 BsAb therapy.

## Case presentation

**Figure 1:**
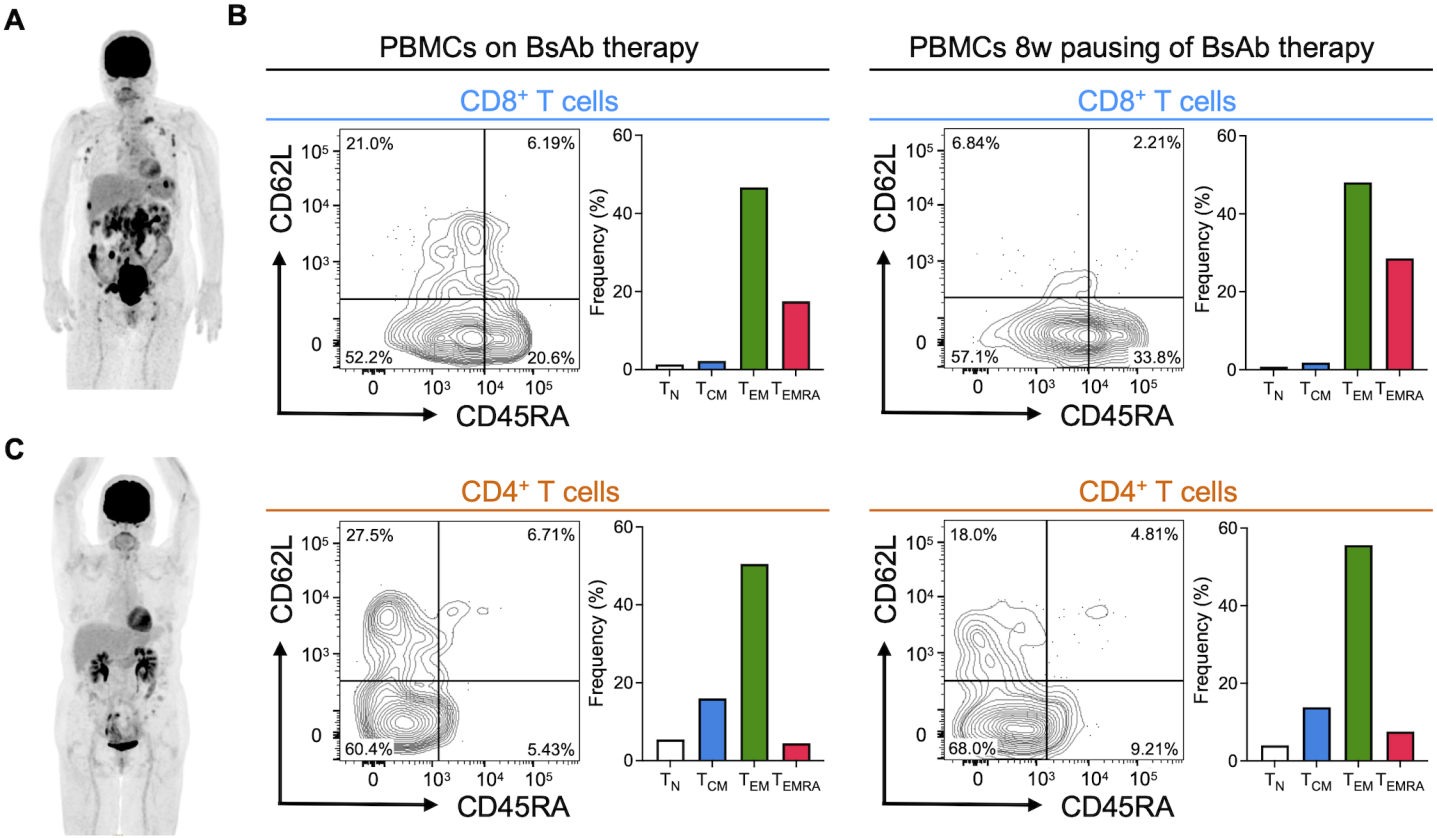
Disease status and T-cell phenotypes of the patient during CD20 BsAb therapy and before CD19 CAR-T cell therapy. **(A)** PET-CT scanning at diagnosis. **(B)** Memory T-cell phenotypes in PBMCs after 6 cycles of BsAb therapy and an 8-week pause of BsAb therapy. CD8^+^ T cells and CD4^+^ T cells were gated and analyzed by flow cytometry. T-cell populations are defined as follows: CD45RA^+^CD62L^+^CCR7^+^ cells as naïve T cells (T_N_); CD45RA^-^CD62L^+^CCR7^+^ cells as central memory T cells (T_CM_); CD45RA^-^CD62L^-^CCR7^-^ cells as effector memory T cells (T_EM_); CD45RA^+^CD62L^-^CCR7^-^ cells as terminally differentiated T cells (T_EMRA_). **(C)** PET-CT scanning just before CD19 CAR-T cell therapy. PET-CT, positron emission tomography-computed tomography.

A patient in their 70s without a past history suffered from constipation and diarrhea. After hospitalization, the patient was diagnosed with DLBCL that had transformed from follicular lymphoma, involving the small intestine as well as surface, mediastinal, and paraaortic lymph nodes (**Figure 1A**). Despite 2 cycles of R-CHOP (rituximab, cyclophosphamide, doxorubicin hydrochloride, vincristine, and prednisolone) chemotherapy, the disease progressed and new intestinal lesions developed. Subsequently, the patient was continuously managed with R-GDP (rituximab, gemcitabine, dexamethasone, and cisplatin) as a second-line chemotherapy, without any marked change in disease status. Therefore, epcoritamab, a BsAb for CD20 x CD3, was administered as T-cell redirection therapy to suppress disease progression. No viral reactivation was observed during the BsAb therapy. As positron emission tomography-computed tomography (PET-CT) revealed complete metabolic response after 3 cycles of epcoritamab, the patient was referred to our hospital for possible CD19 CAR-T cell therapy.

**Figure 2:**
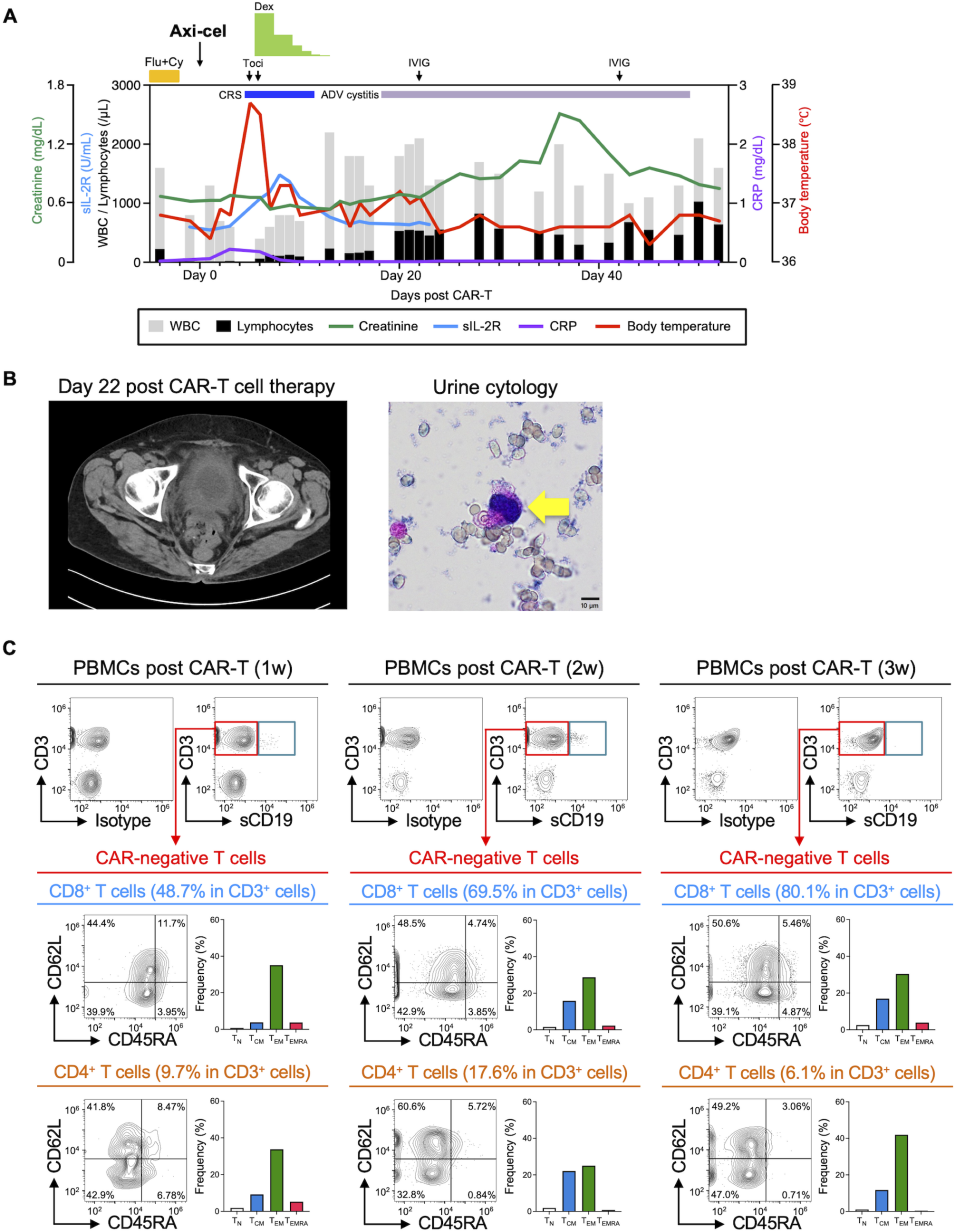
Clinical outcomes and chronological T-cell characteristics of the patient treated with CD19 CAR-T cells following CD20 BsAb therapy. **(A)** The clinical course of the patient after CD19 CAR-T cell therapy. Gray and black bars show the numbers of WBCs and lymphocytes. Red, deep green, blue, and purple indicate body temperature, the values of creatinine, sIL-2R, and CRP, respectively. **(B)** CT scanning shows the bladder on Day 22 after CD19 CAR-T cell therapy (left). Inclusion body identified by urine cytology is indicated by a yellow arrow (right). **(C)** T-cell characteristics in PBMCs after CD19 CAR-T cell therapy. CD19 CAR-T cells were detected by sCD19 dimer. sCD19-negative bystander CD3^+^ T cells were gated on red rectangles to analyze their memory CD8^+^ and CD4^+^ T-cell phenotypes. The definitions of memory T cells are given in **Figure 1B**. Flu, fludarabine; Cy, cyclophosphamide; Dex, dexamethasone; Axi-cel, axicabtagene ciloleucel; ADV, adenovirus; IVIG, intravenous administration of immunoglobulin; Toci, tocilizumab; CRS, cytokine release syndrome; WBC, white blood cells; sIL-2R, soluble IL-2 receptor; CRP, C-reactive protein; sCD19, soluble CD19.

At this point, the patient had received 6 cycles of epcoritamab in total, but suffered from abdominal pain due to local intestinal scarring after treatment. Analysis of the patient’s peripheral blood mononuclear cells (PBMCs) demonstrated predominance of effector memory T cells (T_EM_) and terminally differentiated T cells (T_EMRA_) (**Figure 1B**, left). Based on these findings, our team decided to temporarily pause CD20 BsAb therapy to allow rejuvenation of T-cell activity (9) and improve the patient’s condition by surgical resection of local intestinal stenosis. Although a resting period of 8 weeks appeared not to change the T-cell memory phenotypes (T_EM_/T_EMRA_) of the PBMCs (**Figure 1B**, right), we collected the patient’s PBMCs by apheresis, allowing successful manufacture of CAR-T cells using axi-cel. After the patient had maintained complete metabolic response after one cycle of bridging chemoimmunotherapy with polatuzumab vedotin, rituximab, and bendamustine (**Figure 1C**), the patient then underwent CD19 CAR-T cell therapy in combination with lymphodepletion using cyclophosphamide and fludarabine. The patient’s treatment schedule and laboratory data just before CAR-T cell therapy are shown in **Supplementary Figure 1** and **Supplementary Table 1**. As shown in **Figure 2A**, Grade 1 CRS with fever (38.0 °C) was managed with tocilizumab (8 mg/kg, twice) and dexamethasone (9.9 mg x 4 for 2 days, 6.6 mg x 3 for 3 days, and tapered gradually). Despite maintenance of a normal immunoglobulin G level (452 mg/dL) after CAR-T cell therapy, the patient suffered hematuria and pyuria with renal dysfunction on Day 18. Computed tomography (CT) scan and urinalysis revealed bladder wall thickening and the presence of inclusion bodies in the urothelial cells without bacteria (**Figure 2B**). Further viral screening also demonstrated high copy numbers of adenovirus DNA (4.0x10^8^ copies/μL) but no copy number elevation for BK virus and cytomegalovirus (CMV) DNA. Additional human immunoglobulin (20 g x 1 and 30 g x 1) supplementation and sufficient hydration gradually improved the patient’s condition, and currently the patient still remains in complete metabolic response after 9 months of CD19 CAR-T cell therapy.

## Discussion

In this case, CD19 CAR-T cell therapy following CD20 BsAb therapy successfully induced complete response for relapsed/refractory DLBCL. Some translational studies have mentioned the importance of T-cell recovery for improvement of responses during BsAb therapy (13) and before CAR-T cell therapy (14). Here, our team decided to pause BsAb therapy for 8 weeks, which may be long enough to allow collection of rejuvenated T cells for CAR-T cell therapy (9), although this possibility has been debatable. The patient’s T-cell phenotypes determined using CD45RA/CD62L/CCR7 before and after BsAb pause appeared not to change (**Figure 1B**), suggesting the need for further assessment using additional memory markers such as TCF7 and CD127 in combination with others. However, CD19 CAR-T cells were successfully manufactured, and their administration led to proper development of central memory T cells (T_CM_) and T_EM_ in the patient’s peripheral blood (**Figure 2C**). In this patient, CD19 CAR-T cells in PBMCs were not detectable by flow cytometry at 3 weeks after administration using soluble CD19 dimer (15, 16); therefore, we analyzed the CAR-T cell phenotypes at 1 and 2 weeks, respectively (**Figure 2C** and **Supplementary Figure 2**). The frequency of CAR-T cells peaked at 2 weeks, when CD3^+^CD4^+^ CAR-T cells rather than CD3^+^CD8^+^ CAR-T cells were dominant, in contrast to bystander T cells (**Figure 2C** and **Supplementary Figure 2**). Interestingly, CAR-T cells mostly showed T_EM_ cell phenotypes at 1 week and T_CM_ cell phenotypes at 2 weeks, together with bystander T cells. Although further investigations are necessary, these results appear to suggest that, in the body of this patient, CAR-T cell and bystander T-cell responses differed from each other, and successful memorization of CAR-T cells together with bystander T cells occurred.

Viral hemorrhagic cystitis can be caused by reactivation of latent infection with extant adenovirus, BK virus and others (17). Because T-cell responses are required for efficient suppression of such viral reactivation, such episodes are well documented after allogeneic stem cell transplantation (17–19). Viral hemorrhagic cystitis due to B-cell suppression after CD19 CAR-T cell/CD20 BsAb therapy requires caution; however, there have been a few reports of adenoviral infection after CAR-T cell therapy (**Supplementary Table 2**) (20–22). To our knowledge, it has not been reported after BsAb therapy so far. To discuss the mechanisms involved, we monitored T-cell phenotypes among PBMCs for 3 weeks after CD19 CAR-T cell therapy. Importantly, memory phenotypes among both CAR-negative CD3^+^CD8^+^ and CD3^+^CD4^+^ bystander T cells appeared to differ completely from those obtained before CAR-T cell therapy (**Figures 1B**, **2C**), suggesting recovery of T-cell populations including not only CAR-negative T cells in the CAR-T cell product, but also residual/newly growing T cells after lymphodepletion (16). Furthermore, T-cell receptor (TCR) repertoire sequences before and after CD19 CAR-T cell therapy appeared to differ, as we and others had shown previously (16, 23). Overall, these findings appear to suggest the importance of T-cell recognition via TCRs, i.e., the antigen specificity of TCRs generated by memory-formed T cells in the body after CAR-T cell therapy might change host defenses against viral infections. Adenovirus-specific T cells among the newly-developed memory T-cell population might disappear or become depleted after CAR-T cell therapy, whereas other virus-specific T cells might have been preserved in the present case, thus contributing to suppression of both CMV and BK virus at the point when cystitis appeared. This might also explain why hemorrhagic cystitis was not observed during BsAb therapy, despite the lower level of immunoglobulin G (265 mg/dL, **Supplementary Table 1**). In this patient, we were unable to monitor the presence and frequency of each type of virus-specific memory T cell before and after BsAb/CAR-T cell therapy, or to demonstrate possible expansion of adenovirus-specific T cells after hemorrhagic cystitis, as detailed in a previous study (22). In future, however, comprehensive studies to clarify how memory T cells in the body recognize HLA/peptide complexes presenting viral antigens after BsAb and CAR-T cell therapy might provide further insights into the mechanisms involved.

In summary, we have obtained clinical evidence to suggest that differences in memory T-cell responses may arise during CD20 BsAb therapy and following CD19 CAR-T cell therapy. Even though memory-formed bystander T cells can be successfully developed without immunosuppressant medication after CAR-T cell therapy, there is still a need for caution regarding unexpected adverse events including viral infections which are uncommon complications in the presence of hypogammaglobulinemia. Against a background that has focused on the association of endogenous bystander T-cell expansion with therapeutic outcome after CAR-T cell therapy (23), our findings appear to provide an important opportunity to reconsider antigen-specific bystander T-cell responses after CAR-T cell therapy.

## Data Availability

The original contributions presented in the study are included in the article/**Supplementary Material**. Further inquiries can be directed to the corresponding authors.
