# Supplementary material for: Case Report: Compromised response of memory-formed bystander T cells after CD19 CAR-T cell therapy following CD20 bispecific antibody therapy
Source: Front Immunol. 2026 Apr 22;17:1756756. doi: 10.3389/fimmu.2026.1756756 (PMC13146880; doi:10.3389/fimmu.2026.1756756)
Supplement: Supplementary file 1 [file SupplementaryFile1.pdf]

Case Report: Compromised response of memory-formed bystander T cells after CD19 CAR-T cell therapy following CD20 bispecific antibody therapy

Authors: Yuya Masuda[1], *Junichi Kato[1], *Tatsuya Konishi[2], Takatsugu Honda[3], Masaki Maruta[1], Natsumi Kawasaki[1], Meika Matsumoto[1], Koji Oka[1], Kento Mori[1], Shogo Nabe[1], Yukihiro Miyazaki[1], Etsuko Matsubara[4], Shingo Urata[4], Shingo Kinnami[3], Yasukazu Doi[2,3], Yasunori Takasuka[3], Jun Yamanouchi[2], †Toshiki Ochi[1,5], and †Katsuto Takenaka[1]

Affiliations:

[1]Department of Hematology, Clinical Immunology, and Infectious Diseases, Ehime University Graduate School of Medicine, Toon, Ehime 791-0295, Japan; [2]Division of Blood Transfusion and Cell Therapy, Ehime University Hospital, Toon, Ehime 791-0295, Japan; [3]Department of Clinical Laboratory, Ehime University Hospital, Toon, Ehime 791-0295, Japan; [4]Department of Internal Medicine, Matsuyama Red Cross Hospital, Matsuyama, Ehime, 790-8524, Japan; [5]Division of Immune Regulation, Proteo-Science Center, Ehime University, Toon, Ehime 791-0295, Japan.

*These authors contributed equally to this work.

†Corresponding authors:

Toshiki Ochi, M.D., Ph.D.

Department of Hematology, Clinical Immunology and Infectious Diseases, Ehime University Graduate School of Medicine, Shitsukawa, Toon, Ehime 791-0295, Japan; TEL: +81-89-960-5296, FAX: +81-89-960-5299, E-mail: ochi.toshiki.eg@ehime-u.ac.jp

Katsuto Takenaka, M.D., Ph.D.

Department of Hematology, Clinical Immunology and Infectious Diseases, Ehime University Graduate School of Medicine, Shitsukawa, Toon, Ehime 791-0295, Japan; TEL: +81-89-960-5296, FAX: +81-89-960-5299, E-mail: takenaka.katsuto.hy@ehime-u.ac.jp

Running head: Antigen specificity of bystander T cells after CD19 CAR-T cell therapy following CD20 BsAb therapy

Key words: Diffuse large B-cell lymphoma (DLBCL), Chimeric antigen receptor (CAR)-T cells, Bispecific antibody (BsAb), Bystander T cells, Adenoviral cystitis

Supplementary methods

Cells

Peripheral blood mononuclear cells (PBMCs) collected from the patient were stored in the vapor phase of liquid nitrogen until use. The series of experiments using human samples was approved by the ethical review board of Ehime University, and written informed consent was obtained from the patient for further *in vitro* analyses.

Flow cytometry

The following antibody cocktails were used for sample staining (1, 2). FITC anti-human CD3 (clone SK7), PC5 anti-human CD8 (clone B9.11), APC-Cy7 anti-human CD4 (clone RPA-T4), APC anti-human CD45RA (clone HI100), PE-Cy7 anti-human CCR7 (clone G043H7), and BV421 anti-human CD62L (clone DREG-56) mAbs were employed for staining of T cells among PBMCs. In addition, the soluble form of CD19 in which the extracellular domain of CD19 was fused with an SGSG sequence and 6xhistidine (his) tag at the C-terminus was generated and mixed with PE anti-his mAb (clone GG11-8F3.5.1), then used as a soluble CD19 dimer for detection of CD19 CAR-T cells, as described previously (1, 2). All samples were analyzed using a Gallios flow cytometer (Beckman Coulter) and FlowJo version 10.9.0 software (Beckton Dickinson).

References

1. Ochi T, Maruta M, Tanimoto K, Kondo F, Yamamoto T, Kurata M, et al. A single-chain antibody generation system yielding CAR-T cells with superior antitumor function. *Commun Biol.* (2021) 4:273. doi: 10.1038/s42003-021-01791-1
2. Kato J, Konishi T, Honda T, Maruta M, Nabe S, Masuda Y, et al. Bystander CAR-CD8+ T cells in a CAR-T cell product can expand and enhance the antitumor effects of a bispecific antibody. *J Immunother Cancer.* (2025) 13:e011690. doi: 10.1136/jitc-2025-011690

Supplementary Figure 1

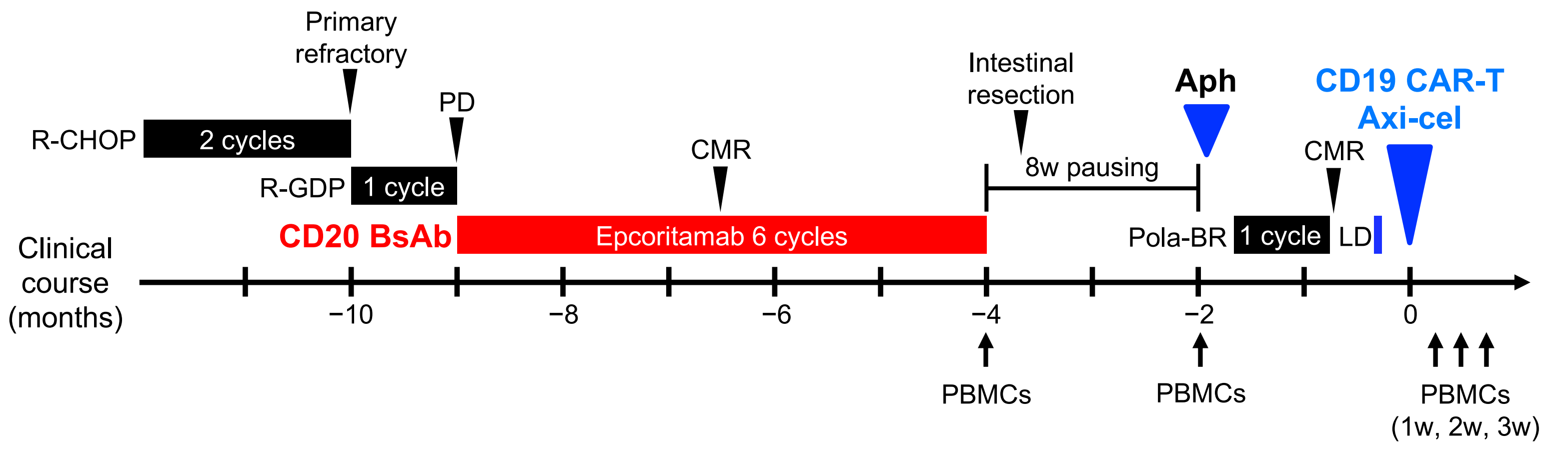

Supplementary Figure 1. Treatment flow for the patient during chemoimmunotherapy and T-cell redirection therapy.

Details of chemoimmunotherapy (black bar), T-cell redirection therapy using CD20 BsAb (red bar), apheresis (blue arrowhead), lymphodepletion (blue bar), and CD19 CAR-T cells (blue arrowhead) are depicted at the top of the horizontal arrow indicating the timeline, respectively. Observed clinical events are also shown with black arrowheads. Timing of PBMC sampling (black arrows) is displayed at the bottom. R-CHOP, rituximab, cyclophosphamide, doxorubicin hydrochloride, vincristine, and prednisolone; R-GDP, rituximab, gemcitabine, dexamethasone, and cisplatin; Pola-BR, polatuzumab vedotin, bendamustine, and rituximab; Aph, apheresis; LD, lymphodepletion; Axi-cel, axicabtagene ciloleucel; PD, progressive disease; CMR, complete metabolic response.

Supplementary Figure 2

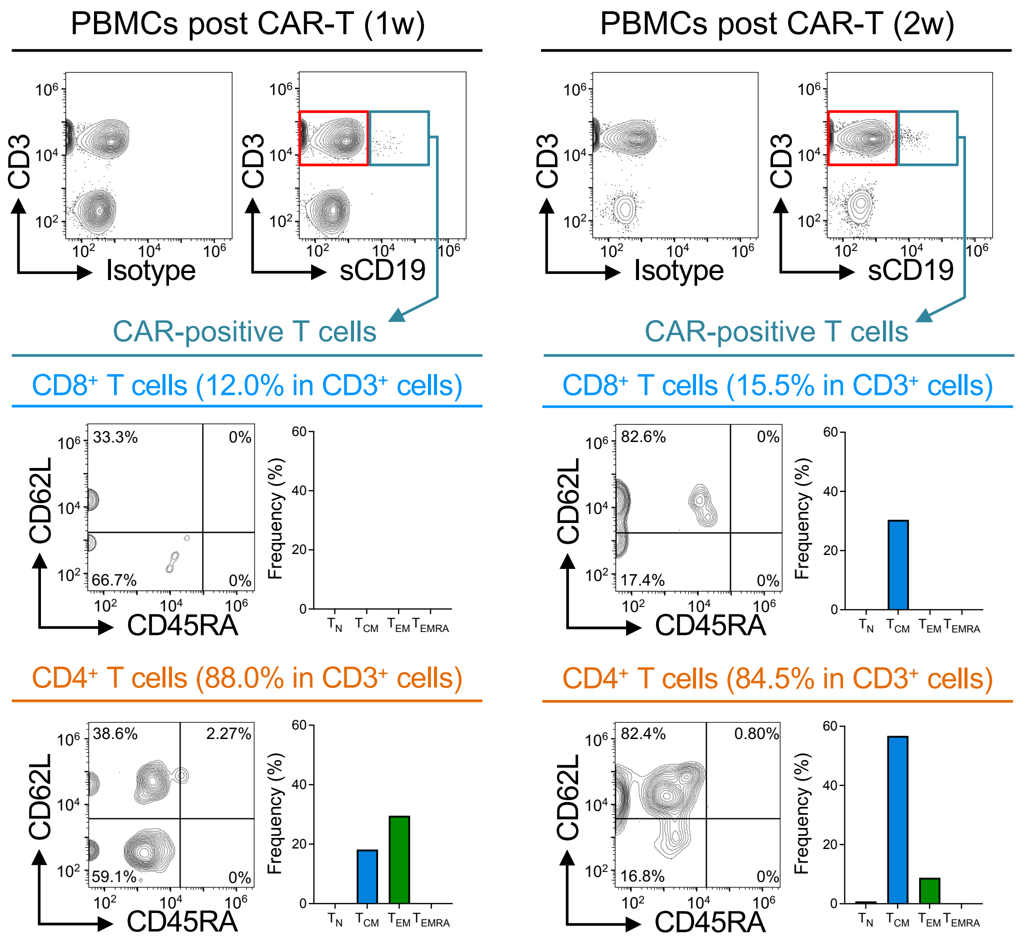

Supplementary Figure 2. Chronological CAR-T cell characteristics of the patient treated with CD19 CAR-T cells following CD20 BsAb therapy.

Characteristics of CAR-T cells in PBMCs after axi-cel administration. CD19 CAR-T cells were detected by sCD19 dimer. sCD19-positive $CD3^+$ T cells were gated on dark blue rectangles to analyze their memory $CD8^+$ and $CD4^+$ CAR-T cell phenotypes. Frequency of $CD3^+CD8^+$ CAR-T cells at 1 week were too low for analysis of their memory phenotypes. The definitions of memory T cells are given in Figure 1B.

Supplementary Table 1

Laboratory tests just before CAR-T cell therapy

WBC	1200	/μL	TP	5.8	g/dL	UA	3.2	mg/dL
Stab	2.5	%	Alb	4.1	g/dL	CK	117	U/L
Seg	26	%	T.Bil	0.5	mg/dL	AMY	84	U/L
Lym	33	%	D.Bil	0.3	mg/dL	CRP	0.1	mg/dL
Mono	33	%	AST	18	U/L	sIL-2R	623	pg/mL
Eosino	5	%	ALT	14	U/L	β_2MG	2.7	mg/dL
Baso	0.5	%	LDH	213	U/L	IgG	265	mg/dL
RBC	3.29	10^6/μL	ALP	132	U/L	IgA	32	mg/dL
HGB	9.4	g/dL	γ-GTP	143	U/L	IgM	7	mg/dL
HCT	28.7	%	Na	143	mEq/L	APTT	26	sec
MCV	87.2	fL	K	4.2	mEq/L	PT	126	%
MCH	28.6	pg	Cl	107	mEq/L	Fib	302	mg/dL
MCHC	32.8	%	BUN	20	mg/dL	ATⅢ	118	%
Ret	26	‰	Cre	0.6	mg/dL	FDP	2.8	μg/mL
PLT	17.3	10^4/μL	Ca	9.9	mg/dL			

Supplementary Table 2

List of cases of adenoviral infection after CAR-T cell therapy

References	Years	Disease	CAR-T target	Infection Organ	Virus	Onset after CAR-T	Treatment	Outcome
Our's report	2025	DLBCL	CD19	Cystitis	Adenovirus	18 days	IVIG	Cure
20	2020	DLBCL	CD19	Cystitis, Viremia	Adenovirus	2 months	Cidofovir	Cure
21	2023	LBCL	CD19	Cystitis	Adenovirus	Within 30 days	N.A.	N.A.
22	2024	MM	BCMA	Systemic	Adenovirus	2 months	IVIG, Cidofovir	Cure
